# Longitudinal online diaries with dental practitioners and dental care professionals during the COVID-19 pandemic: A trajectory analysis

**DOI:** 10.3389/froh.2022.1074655

**Published:** 2022-12-22

**Authors:** Laura Beaton, Jennifer Knights, Lorna Barnsley, Mariana Araujo, Jan Clarkson, Ruth Freeman, Linda Young, Siyang Yuan, Gerry Humphris

**Affiliations:** ^1^ NHS Education for Scotland, Edinburgh, United Kingdom; ^2^ School of Dentistry, University of Dundee, Dundee, United Kingdom; ^3^ School of Medicine, University of St Andrews, St Andrews, United Kingdom

**Keywords:** COVID-19, dentistry, qualitative research, longitudinal studies, trajectory analysis, burnout, mental health, wellbeing

## Abstract

**Introduction:**

The COVID-19 pandemic resulted in a series of significant changes and adjustments within dentistry, as dental professionals dealt with temporary closures of dental practices, increased use of personal protective equipment, a reduction of clinical procedures, and extensions to training programmes. Recent research has illustrated the impact of the pandemic on the dental profession, indicating that many dental professionals felt emotionally exhausted and experienced significant uncertainty and anxiety. This qualitative study aimed to understand how these experiences and emotions changed over the course of six months, in dental trainees and primary dental care staff in Scotland.

**Methods:**

A longitudinal diary study was conducted (June—December 2020) with dental trainees and primary dental care staff. The diary asked respondents to answer three questions related to their emotional exhaustion, on a weekly basis. There was also an open question asking respondents to describe any significant issues or concerns they had experienced during the preceding week because of the impact of the COVID-19 pandemic on their work or training. This qualitative data was explored using a trajectory analysis approach to determine specifically changes over time.

**Results:**

The trajectory analysis revealed several key concerns prevalent amongst respondents, and how they fluctuated over the six months. Concerns included: the impact of the pandemic on respondents' future careers and on dentistry more generally; adapting to new working environments; the impact on their patients' dental treatment and oral health; the impact on their health and wellbeing; financial considerations and adjusting to new safety measures as part of the remobilization of dental services.

**Discussion:**

In the second half of 2020, as the UK was adjusting to the introduction of new COVID-19 safety measures in everyday life, the dental profession were grappling with significant changes to their working environment, including PPE, redeployment, use of aerosol generating procedures (AGPs), and timelines for re-opening practices. This longitudinal diary study has shown some parts of the dental profession in Scotland expressed very varied and personal concerns and anxieties related to COVID-19. Respondents' candor in their diary entries revealed explicit, frequent and high levels of uncertainty and worry related to their training and career. Collectively, the data corpus highlighted the emotional toll these anxieties have taken on the dental professions in Scotland.

**Conclusion:**

These findings demonstrate the need for (a) increased provision of mental health and wellbeing support services for dental staff and (b) the study of the linkage between organization of pandemic management to the working practices of staff delivering services. Interventions, at various levels, should take into consideration the fluctuating nature of dental professionals' concerns and anxieties over time, to address both immediate and longer-term issues.

## Introduction

The detrimental impact of the COVID-19 pandemic on the mental health and wellbeing of dentists and dental care professionals has been demonstrated across several studies internationally (1–3). However, while these cross-sectional studies make an important contribution to developing the evidence base around mental health in dentistry, they need to be complemented by longitudinal designs that allow for a more in-depth exploration over time.

In Scotland the “CAREER” project was established during the early months of the pandemic to develop an understanding of the pandemic's effect on anxiety, feelings of uncertainty and preparedness of dental practitioners and dental care professionals working in primary care dental teams (4). At baseline, 27% of participants reported depressive symptoms (compared with 18% in a population-based cohort under normal conditions) and 55% as experiencing emotional exhaustion (5). The longitudinal component of the project focused on emotional exhaustion as a precursor of burnout, especially the development of burnout over time. Analysis of the quantitative longitudinal data, collected in the form of weekly diaries over a six-month period by a subset of the study population, found that dental staff showed, on average, a 25% deterioration in their wellbeing. Variation between respondents in their fatigue trajectories was significant. These included stability of reporting at low, medium, and high levels of emotional exhaustion. In addition, and most interesting, were the number of respondents who showed remarkable changes in their emotional exhaustion including those who deteriorated as well as some who demonstrated surprisingly, on first inspection, a lowering of fatigue. So, although there was an overall raising of fatigue during the period of study, the variation of response supported the view that staff differed strongly in their ability to manage the emotional demands of treating patients under the constraints of the COVID-19 pandemic (6). However, the quantitative study was limited in not being able to provide detailed explanations of individual changes in their fatigue trajectory. The collection of free responses in the open-ended section of the weekly diary form provided a unique opportunity to explore content and enable the derivation of explanatory factors. This qualitative study aimed, therefore, to understand how respondents' experiences and emotions changed over the course of six months.

This paper presents the qualitative diary data collected in the longitudinal component of the project. The collection of chronologically organized qualitative data about participants' weekly issues, concerns and activities permitted a detailed exploration of key stressors for each individual over time. It also allowed for the consideration of themes across and between different groups of staff, again considering how these changed over time. As far as the authors are aware, this is the first attempt at a qualitative longitudinal diary study in the academic field of mental health and wellbeing of dentists and dental care professionals.

## Methodology

### Study design

This is a qualitative longitudinal study that used online diaries for data collection. Unlike other qualitative methods such as interviews, diaries allow for capturing descriptions and emotions in a relatively spontaneous way, nearer the real time of event occurrence. Proponents suggest that this special claim to immediacy may allow the researcher to get closer to the “truth” of a phenomenon (7). Quantitative approaches to diaries, by focusing upon certain pre-defined factors, may overlook other factors that might be important, neglecting valuable insights participants may have to offer (8). Contrastingly, qualitative diaries permit participants to depict their own priorities, therefore allowing for the capture of meaning and weight that diary respondents themselves can attach to different events and problems in their lives (9).

### Sample and recruitment

All diary respondents had previously participated in a baseline survey conducted by the authors as part of the CAREER project (5). A non-probability convenience sample was used to recruit participants for the survey, as detailed in Humphris et al. (5). Dental trainees were invited to participate in the survey *via* their training programmes in mid-June 2020; primary care dentists and dental care professionals were contacted in August 2020 *via* the NHS Education for Scotland (NES) Portal, an online course booking system where dental professionals can opt in to receive marketing communications. The survey provided respondents with the option to volunteer to keep a brief weekly diary for the duration of the six-month study, related to their emotional state and routine. Eighty-two respondents from the survey volunteered to take part in the diary study.

Validity of diary entries was established through a two-part process. Firstly, any diaries with less than 4 submissions over the period of study were omitted. This aligned with the validity criterion established in the quantitative study (6). Secondly, those that provided less than two qualitative entries, thereby preventing comparison over time, were omitted. A further diary was omitted due to the participant being an undergraduate at the time of diary commencement and therefore not within the scope of the analysis.

### Data collection

Diaries were collected between July and December 2020^1^. Diary respondents were contacted by email with a link to access the online diary form made available on the survey platform Questback. The form asked participants to select their response to three items drawn from the emotional exhaustion subscale of the Maslach Burnout Inventory (10), and then in an open text box describe any significant issues or concerns they had experienced during the preceding 7 days because of the impact of the COVID-19 pandemic on their work (or training). This paper presents findings from the qualitative data collected *via* the open text box; the quantitative results have been reported elsewhere (6).

The research team asked respondents to try and complete their diary entry every Friday, or as near as possible to the close of each working week, whilst events were still fresh in their minds. The project administrator (LBa) reviewed diary completion rates at the beginning of each working week and sent reminders where entries were outstanding. In cases where three reminders had been sent yet no further diary entry had been made (in terms of at least completing the three items from the emotional exhaustion subscale) reminders were discontinued and the participant not contacted again unless they recommenced completing their diary independently.

### Data analysis

The qualitative diary responses were analyzed using *trajectory analysis*, a type of analysis which, through the coding of data into time-ordered sequential matrices, allows analysis to focus on changes over time for individuals and small groups of individuals (11). The method was adapted into 4 key steps:

1. Familiarize with data through reading and re-reading.
2. Code data into matrices set up for each participant, capturing themes along the y-axis and time along the x-axis.
3. Organize individual themes into a large matrix per professional group, capturing cross-cutting themes along the y-axis and individuals along the x-axis. Identify any new conceptual groupings emerging from the progression of the second matrix.
4. Conduct final data analysis from the second matrix in which codes focus on change over time (or not, where concepts remain stable), referencing back to the individual matrices where specific examples are required.

Qualitative content analysis provided the theoretical basis for theme development (12) and Microsoft Excel was used to code data into matrices. Professions were grouped for step 3 as follows:

a. Trainees: Vocational Dental Practitioner (VDP), Vocational Dental Therapist (VDT), Dental Core Trainee (DCT), Specialty Registrar (StR), Trainee Dental Nurse, Trainee Orthodontic Therapist.
b. Dentists: Dentist (GDS), Dentist (PDS).
c. Dental care professionals (DCPs): Dental Nurse, Hygienist, Therapist.

Data analysis was conducted by LBe and JK; regular discussion and reviewing of themes acted to ensure trustworthiness in terms of credibility (13, 14).

The stories in Boxes 1–3 were created through a narratological process of turning the diary data into stories (15). The purpose of these storied accounts is to allow the reader to engage with the full affective power of the unique accounts provided by individual participants, while also reflecting on the broader outcomes of the thematic analysis. Three diaries were selected for this purpose, one from each professional grouping. The data was analysed for story elements such as characters, settings, actions, and resolutions and then reorganised into story form: the process of emplotment and the forming of each story as a coherent whole must be understood as an act of interpretation (16). Chronology was already supplied through the longitudinal nature of the data collection.

### Ethical considerations

The study involved human participants and as such was reviewed and approved (18th May 2020) by the University of Dundee Nursing and Health Sciences and Dentistry Research and Ethics Committee (Reference: UOD/SDEN/STAFF/2020/013- Freeman). The participants provided their written informed consent to participate in this study.

## Results

Following the application of validity criteria, 56 diaries were considered valid for qualitative analysis from a total of 82 completed diaries.

### Trainees

Twenty-three trainees completed diary entries between July and December 2020 as part of the CAREER study. Diaries from 14 of these respondents were considered valid for the purposes of trajectory analysis (6 VDPs, 1 VDT, 2 DCTs, 3 StRs, 1 Trainee Dental Nurse, and 1 Trainee Orthodontic Therapist).

#### Impact on training and future career

Of concern to many respondents was the impact of COVID-19 on their training and their future career opportunities.

Four respondents (1 VDT, 1 StR, 1 Trainee Dental Nurse, 1 Trainee Orthodontic Therapist) reported consistent anxiety throughout the data collection period from academic stressors, particularly preparing for exams. These respondents' diary entries showed continuous activities relating to exam preparation. The Trainee Dental Nurse found the time and energy to study for exams and re-sits to be an ongoing challenge:

*“This week I was worried about upcoming exam. I had not had chance to study as hard as I wanted, because I felt too tired and could not concentrate… It is weird how draining it is to return in full time job and how unsuccessful I have been balancing work and studies [the] past couple weeks. I must find [a] way how to catch up with tasks.”* (Trainee Dental Nurse, Week 7)

Over time, the stress related to preparing for exams shifted towards an increasing anxiety about exams being cancelled because of COVID-19 restrictions.

“*Exams hopefully still going ahead next month but feeling worried at the moment because of rising cases in my area and worried that the exam will be cancelled again*.” (Trainee Orthodontic Therapist, Week 15)

The impact of COVID-19 on respondents’ training was a constant concern for almost all respondents, although this lessened for some by the end of the data collection period when they were able to gain more experience. Concerns related, in particular, to a lack of opportunity to develop and maintain clinical skills, and the potential to become deskilled. This was also associated with an increasing lack of confidence. Related to this concern about clinical skills, several respondents were also worried about meeting their training requirements and anxious about the lack of available opportunities to complete assessments. One StR writes in week 2:

“*My supervisor has been extremely busy this week dealing with service planning etc. and therefore there is limited time for training or carrying out workplace based assessments*.” (StR, Week 2)

They highlight the same issue almost every week for 7 weeks until, for them, the situation starts to improve:

“I *managed to get some workplace based assessments completed and it was a good week.*” (StR, Week 9).

These training issues were found across the data collection period for most respondents. For several respondents, these anxieties decreased towards the end of the data collection period by reference to gaining more relevant experience. One StR was initially excited about the new opportunities presented by the change in working patterns, but this feeling reversed to match the worry felt by the other respondents.

Concerns about job and financial security were found periodically increasing in frequency as respondents approached the end of their current training programme. These comments were made by seven respondents, representing most of the trainee professional groups (VDP, DCT, StR, Trainee Dental Nurse). A common thread was uncertainty about what would happen at the end of the specific training period. For VDPs, the primary anxiety was what would happen in the period between their vocational training year ending and them starting another position (either another training programme or in practice). These feelings were reflected in diary entries for many weeks across the data collection period, with VDPs feeling stressed, miserable, and worried about the uncertainty. These respondents explicitly reported worries about their financial security, and delays with payments.

Box 1 Sarah’s* Story.

Sarah is in her mid-20s and training to work as an Associate in General Dental Practice. When she submits her first diary entry on 9^th^ July she is just back in clinic after 4 months and feels that her confidence in making decisions has taken a big drop. August is approaching fast, meaning the end of her training: how will she cope without her Trainer at hand? She doubts herself, questioning her abilities: “Decoronated lower molar, difficult extractions, was it my technical ability due to lack of practice or was the tooth always going to break?” Finances are also a worry: how is she going to make any money in practice? Six years in training and her parents may need to help with the rent, this is not at all what she had expected!

Come mid-August her frustration is mounting. She has the knowledge, skills and equipment to get people out of pain and prevent dental disease, but she isn’t allowed. It seems to her that “money/access” is being chosen over patient need and she questions the morality and the ethics of the situation. Patients are frustrated, waiting lists are growing, and she is annoyed that years of preventative dentistry and education have been completely reversed. She is now struggling financially, with fuel costs and lab bills she is actually working at a loss. This leaves her feeling drained. She even has nightmares about patients threatening lawsuits over loose teeth (although the Headspace App does help with the stress before bed). Although she is nervous of the idea of doing AGPs again, including wearing PPE for long periods of treatment time, she hopes for a return to more routine care soon.

Sarah’s final diary entry is on 18^th^ September. She has been paid for the first time since July and this has made her feel a bit more motivated about working. The routine of work and a sense of normality compared to doing phone triage has made her feel better. However, the ethical questions persist: “I”m in a moral dilemma not being able to provide routine/preventative care on the NHS. We have been offering private fills etc. but the financial inequality doesn’t sit well with me at all.” The future does not look good, she sees “no sign of release” and fears for her professional future and those of her peers: will they choose alternative careers? Will she? She ends her diary on a bleak note: “this job is not satisfying and not enjoyable”.

*Sarah is a pseudonym.

“*Preparing for work as an associate without the comfort of a monthly salary and a huge amount of uncertainty regarding financial support and security has contribute[d] to some increasing anxieties as I come to the end of vocational training.*” (VDP, Week 4)

Similarly, two DCTs expressed feelings of uncertainty regarding their futures, with one respondent reporting constant stress about this, along with the pressure of submitting job applications, over the course of five weeks of their diary entries.

#### New working environment

Fluctuating levels of anxiety were found related to the need to adapt to new working environments and working patterns during the pandemic. These anxieties were related to adapting to busier/quieter workloads, taking on new responsibilities, and learning new procedures and processes, including wearing Personal Protective Equipment (PPE). These anxieties were particularly evident at the initial stages of data collection.

The diary of one VDP shows how their concerns and anxieties around PPE changed over time: initially they were concerned about availability; this changed into nervousness about discomfort wearing masks while talking to patients. Several weeks later the respondent reported having their mask fitting.

Three respondents expressed anxiety about ambiguities regarding processes and procedures they were expected to follow, including the changing COVID-19 rules for dentistry. Communication between the government, health boards and dentists around procedural changes was also cited as a concern. For two respondents, this anxiety was expressed early in the data collection period and was related to stress about learning new protocols and frustration with the ambiguities. However, for one respondent, this anxiety lasted for several weeks, and their motivation to keep up to date with changes fluctuated during this time.

Adaptations had to be made for some respondents regarding their working environment, with one StR reporting working from home and another StR discussing for several weeks the challenges of juggling their professional role with childcare:

“*Continuing to have childcare issues requiring use of annual leave and loss of time at work to cover childcare needs, important meetings scheduled for non-working days and having to dial in with a baby on my lap.*” (StR, Week 4)

Three respondents noted in their diaries that they had had the opportunity to get some respite from work demands by taking annual leave; for some this helped reduce anxiety, but for others it made no difference.

Concerns regarding workload were raised by 7 respondents, including VDPs, StRs and the VDT. Several respondents noted that their workload was quiet and slow in the early weeks of data collection, with one expressing anxiety about the reduction in the number of patients. Reduced workload remained an issue for one respondent sixteen weeks later, but the other respondents experienced fluctuating levels of busyness or increasingly and then consistently heavy workloads. One respondent reported that they initially felt uncomfortable being redeployed, although they described enjoying their role 19 weeks later. Similarly, another respondent, who reported having a heavy workload throughout the data collection period felt their workload was taking them away from clinical dental work.

#### Health and wellbeing

Respondents used their diary entries to reflect on their own mental health and wellbeing. Four respondents discussed the level of support they felt they were receiving from supervisors and colleagues. Three of these felt continuously supported through the data collection period.

“*Although surgical/clinical hands-on activity is still limited, I can clearly see the efforts to which my department lead, [Educational Supervisor] and [Training Programme Director] are going to prioritize my training and ensuring I get as much operating as possible.*” (StR, Week 9)

One respondent, a VDP, explained that they were worried about a potential lack of support once they moved into an associate role, having relied on their VT trainer to support them when they felt a loss of confidence due to their lack of clinical experience.

Concerns around mental health and wellbeing were raised by four respondents, with wellbeing appearing to fluctuate over the data collection period. Respondents reported trouble falling asleep, nightmares about work, dealing with upsetting personal news and traumatic events in their practice, and constant feelings of tiredness, low motivation, and exhaustion. By Christmas one StR described “*feeling close to burning out*” and “*not hopeful of situation improving any time soon*” (StR, Week 24).

Concerns about COVID-19 were expressed by two respondents, in relation to personal safety and the national response to the pandemic. One respondent initially expressed worry about the risk of contracting COVID-19. This worry reduced over time when they returned to work, but then re-emerged two months later when case numbers were rising. The second respondent initially felt optimistic for the country's recovery, but this positivity was superseded later in the data collection period with frustration over people not adhering to the COVID-19 safety rules and restrictions.

#### Treating patients

Several respondents noted concerns about treating patients. Two respondents reported early in the data collection period that they were frustrated and anxious because they were dealing with patients who were “*demanding*” and “*hostile*”; this frustration initially lessened for both. One respondent, however, reported these feelings returning several weeks later.

In addition, two other respondents suggested they were experiencing moral or ethical dilemmas related to the provision of care for their patients. One respondent expressed frustration with not being able to provide the care they felt their patients needed, and that the NHS overall was failing to meet patient need. The second respondent initially reported feeling concerned about what treatments they could offer, but this changed over time into stress about explaining treatment restrictions to patients. Later on, this respondent began to feel stressed about what the restrictions meant for their professional responsibility and the duty of care they have for their patients.

#### Change over time

Overall, the themes that emerged from the trainee diaries were often negative and remained constant or increased over time, e.g., an increase in worry about job security, an increase in moral or ethical dilemmas about the impact on the oral health of patients, and constant academic stress. In some instances, the themes reflected a positive change over time, e.g., frustration with patients decreased, and the impact on training decreased for some trainees towards the end of the data collection period. Other themes fluctuated across the data collection period, often reflecting the ever-changing Covid-19 situation across the country: there were fluctuating concerns about the risk of Covid-19, the use of PPE, and the impact on workload.

### Dentists

Thirty-four dentists completed diary entries between September and December 2020. Of these, 22 matched the inclusion criteria (20 GDS dentists, 2 PDS dentists).

#### Impact on future career and finances

Six respondents (5 GDS dentists, 1 PDS dentist) used their diary entries to report that they were considering changing jobs or leaving their current practice. One GDS dentist moved into the PDS during the data collection period, seeking better job security. For two respondents their diary entries changed over time to reflect their considerations, progressing from expressing an initial desire to change their circumstances to actually making a significant change through retiring or selling their practice. In both instances this was followed by a sense of relief and a reduction in stress. For another GDS dentist, while there were several mentions of their thinking about leaving dentistry, this changed over time. By the end of the data collection period, they were feeling more positive and grateful for their work.

Six GDS dentists expressed concerns about their financial security. For most this consisted of one-off statements. However, for the two respondents that raised this issue more than once, the impact of the pandemic on their finances was a constant worry, with one noting their income had been “*drastically reduced*”.

#### Working environment

Difficulties within the working environment were raised frequently in the dentists' diaries, including issues with staffing in their practices, the challenge of adapting to new ways of working, including PPE, problems with workload and poor morale.

Workload was raised by the majority of respondents (14 GDS, 1 PDS). For those who mentioned their workload more than once, they were either constantly very busy throughout the data collection period, leading to these dentists feeling overwhelmed and exhausted, or their workload increased over time, with the consequence of an increase in reported stress levels.

“*I have a lot of patients to fit into 2 days and prioritising treatment is more stressful than it should be.”* (GDS Dentist, Week 20)

One respondent initially wrote about their feelings of guilt around not being sufficiently busy, but over time this changed to feelings of being overwhelmed with work. Another respondent, while noting that their workload-related stress had increased over time, concluded their diary entries with optimism about the future following their return from the winter break.

Two respondents (1 GDS, 1 PDS) highlighted frustration with staff they managed or supervised, with disagreements over clinical procedures and difficulties in trying to ensure the team were up to date with important information. The impact of the pandemic on other members of the team was acknowledged by three GDS dentists, who used their diaries to describe the high stress levels being experienced by colleagues. In addition, low workplace morale was raised as a concern by two other GDS dentists. For one of these dentists, these feelings intensified over time, with a sense of being underappreciated developing into an increasingly strained relationship with the practice owner.

Covid-19 precautions in dental practices elicited several concerns from diary respondents. Nine GDS dentists used their diaries to discuss issues with wearing PPE; five of these respondents made repeated mention of the subject throughout the data collection period. Two respondents moved from reporting initial discomfort or delays and frustration caused by mask-wearing to becoming more accustomed to it or feeling satisfied when the whole team had been fitted. Another respondent reported ongoing stress and frustration on the topic over the course of six diary entries. Representing another perspective, one dentist initially felt “*exhausted*” due to wearing a mask, yet over time their reflections turned to the sustainability of the PPE going forward.

Additional concerns related to adapting to new ways of working were raised by two GDS dentists. For one, this began with feeling daunted about new procedures and new technology, before later adapting and acknowledging that “*There will be a few hiccups along the way and it will take time for the new timetable to work well.”* Uncertainty around how to adapt to the new working environment was raised by eight GDS dentists and one PDS dentist. The PDS dentist detailed on several occasions their concerns about doing domiciliary visits without being tested for COVID-19. Issues experienced by the GDS dentists included not knowing exactly what treatment they could provide, uncertainty about the Scottish Government guidance, and confusion around patient charges. For some, this uncertainty spanned several weeks' worth of diary entries, remaining constant. For one GDS dentist, this began to turn into feelings of frustration after being “*in limbo”* for several weeks.

Four GDS dentists noted that their practice was experiencing staff shortages. For two, this developed from noting that extra staff were required, to later diary entries detailing the difficulties in practice because of the staff shortages, with staff experiencing significant pressure.

For two dentists in the GDS, the impact of the pandemic on the practice operating as a business was causing concern. One reported feeling under pressure to prevent the business “*going bust*”. The other made several diary entries expressing anxiety about whether their business would survive. Over time this anxiety increased, with the respondent noting that the stress was making the practice staff ill.

#### Health and wellbeing

A range of concerns related to respondents’ health and wellbeing were reported in the diaries. A common anxiety was the ongoing uncertainty around COVID-19; this was raised by nine GDS dentists. This anxiety increased over time for some, and for others remained constant. For those where an increase was observed, often this was prompted by feeling of helplessness, or where a colleague, patient, or family member had tested positive.

Box 2 Keith’s* Story.

Keith is a General Dental Practitioner in his early 50s working in a mixed NHS/Private practice. His first diary entry on 6^th^ October finds him feeling drained from managing the effects on the team of a patient making contact to say they had tested positive for COVID-19 and the subsequent Track and Trace** follow up. The incident has been very disruptive to staff, highlighting different anxiety levels within the team. He is also frustrated, everyone in his practice is working so hard, trying to put patient interests at the centre of practice decision making, yet it seems others “are doing very little and barely opened their doors. We all get the same 80% NHS funding”.

Another staff contact with a positive case arises the following week, creating further anxiety within the team and leading to discussions which expose “flaws” in the central guidance on testing and isolation. Keith considers it to be inevitable that there will be positive cases in the practice while local health board numbers are “very alarming”. While this reinforces the need for universal precautions it is proving exhausting; he is extremely tired from full days wearing PPE. The precautions are also creating a staff shortage within the practice. Such challenges are difficult to manage, as is scheduling the backlog of new patient referrals to the practice. Entering November Keith takes a day off, so he feels less “frazzled” and “slightly buoyed by further remobilization of NHS dentistry”. However, he is still drained and as the month progresses, he comments on how communicating with PPE on at all times is exhausting and how much he needs a holiday. End of November sees him having a week off: “Time without patient pressures, demands and constant PPE has been SO welcome”.

Moving into December and patient expectations seem to be reverting to a pre-pandemic state. This makes maintaining a good level of clinical service with reduced staff, whilst not compromising covid protocols, increasingly difficult. This pressure becomes unrelenting: “I am trying to mentally adjust to the fact that I cannot meet patient demands and expectations. Trying to say no. This is difficult after 30 years of clinical provision where I have been programmed to say yes”.

Managing patient expectations, trying to talk to them from behind his PPE to explain why the service is modified, leaves Keith exhausted. He finds some optimism in the fact a vaccine is now approved: “I cannot begin to tell you how happy I am that there may be a chance of relief from the pandemic conditions. A glimmer of hope”.

As the year closes Keith expects that the financial model will look very different in the long term. He suggests that general dental practice shall need to change, adapt and perhaps accept less profitable practices: “The financial model is changed forever. I feel resigned”. He concludes: “Quite glad I'm in my 50s and not my 20s!”

*Keith is a pseudonym.

**Scottish COVID-19 Contact Tracing Programme

“*Testing for coronavirus has shown a significant increase in cases. There seems to be far more now and I am worried about staff getting ill. I feel constantly on my guard…”* (GDS Dentist, Week 16)

Several other issues affecting respondents’ mental wellbeing were raised in the diaries. One GDS dentist made repeated mention of feeling isolated and the negative effects of being unable to see colleagues outside of their “bubble”. This was a constant throughout their diary entries. Three GDS dentists outlined the advantages of taking time away from work—in all instances they reported feeling less stressed or more refreshed on their return. For another three GDS dentists, getting back into a working routine provoked varying results. Two respondents found comfort in routine: both moved from a state of stress and uncertainty to finding enjoyment in their “*new normal*”. However, another respondent mentioned feeling depressed on several occasions because they found their routine to be mundane:

“*Nothing ever happens, all days are exactly the same, I don’t have anything to look forward to, that's why it is so depressing.”* (GDS Dentist, Week 23)

Stress was also discussed by several respondents. For two GDS dentists, while they used the open text box in the diaries to report their stress levels as being high in their early diary entries, these appeared to reduce over time, with one respondent stating that the roll out of the COVID-19 vaccine had made them feel more positive. However, for three other respondents (2 GDS, 1 PDS), their level of stress increased over time—one reported feeling emotionally exhausted, while another described feeling demoralized and as in despair, describing their situation as “*soul-destroying*”. Two GDS dentists reported physical health issues, including loss of appetite, and sleeping problems.

Concerns related to returning to clinical work were also raised, with one GDS dentist reporting that they felt out of practice, but a few weeks later this feeling had passed, to be replaced with issues about patient scheduling and planning. Another respondent, a PDS dentist, used one diary entry to express their concern about returning to normal practice, noting “*the fear is worsening*”.

#### Frustration with leaders and management

Various frustrations with management were raised by eight respondents (7 GDS, 1 PDS) in their diary entries. Most of these frustrations were raised on one occasion only, and as such there was no opportunity to observe change over time. Pressure from practice management to bring in more money or do extra clinics was described, and complaints made about health boards being slow to act. Two respondents stated that they felt unsupported by their health board. One respondent, a GDS dentist, felt angry with and unsupported by the General Dental Council (GDC). The most frequently raised frustration was with the Scottish Government, namely their announcement during October 2020 that as of 1 November 2020 dental contractors would be able to provide “*a full range*” of dental treatments to NHS patients (17). Five GDS dentists noted in their diaries feelings of frustration, anxiety and stress related to the uncertainty surrounding this announcement, and the fact there had been no advance notice provided to practitioners.

“*I have been feeling more stressed this last week. I believe this is more related with the uncertainty that the last week's announcement from the government and CDO has caused. The news are that from November all the NHS services will resume in dentistry, however there is no clear guidance on this. It is the first time during COVID-19 pandemic that I feel that unsettled.”* (GDS dentist, Week 17)

#### Impact on patients

One of the most frequently raised topics within the dentists' diaries was the impact of the pandemic on patients. This impact was two-fold, evidenced by respondents' concerns about managing patient expectations, and the implications for patients' oral health due to limited treatment options.

Fifteen dentists (14 GDS, 1 PDS) discussed their feelings about patient expectations, patient demands, or patients being rude.

“*The problem is that the treatment we have been bound by under the SDR has been changing constantly throughout the year. Most people accept this but others can become quite angry and frustrated. I suppose that's human nature, everyone will respond differently to the same situatio*n.” (GDS Dentist, Week 16)

There was variation in how these diary entries changed over time; for some the frustration or exhaustion created by patient demands remained constant throughout the data collection period, but for others their concerns increased over time as patient demand for treatment grew, leading one GDS dentist to describe their job as “*fire-fighting*”.

Ten dentists (8 GDS, 2 PDS) expressed concerns about the limited treatment options available to patients. For 6 respondents, this was restricted to one diary entry each on the topic but for some this was an ongoing concern, with frustration, exhaustion and guilt expressed about not being able to provide optimal treatment to patients.

“*I am exhausted speaking with patients as it often involves detailed chat on diagnosing their problem and unfortunately the outcome is often advanced treatment. Extractions are more often surgically as the tooth has been left too long which creates an AGP appointment which impacts on the rest of my day. I am exhausted from emotionally supporting my patients through emergency care which is much harder than when providing routine care.”* (GDS Dentist, Week 22)

For others their concern increased over time or fluctuated between concern for patients and relief when more treatments could be offered. Two dentists (1 GDS, 1 PDS) noted that patients were enquiring about private vs. NHS provision, in light of the treatment limitations.

#### Future of dentistry

Concerns about the future of dentistry were raised by five GDS dentists. These concerns related to the ability to retain staff, a loss of collegiality within the profession, and the payment model. For one respondent, comments about the payment system in dentistry were repeated over several diary entries, starting with concerns about the development of a two-tiered system to later suggestions around a possible new model which would ensure that dentists were paid more money. For another respondent, concerns about the erosion of the dental profession, specifically the feeling of loss of collegiality, increased over time.

#### Change over time

For dentists, the majority of themes that emerged from diary entries remained constant or increased over time. This reflected constant or intensifying anxieties or concerns related to the impact of Covid-19 on respondents' work, including staff morale, job security, financial concerns, patient expectations, and an impact on treatment options for patients. One theme that showed improvement, or a lessening of concern over time, was related to anxieties with wearing PPE. Several other themes, including stress and adapting to new ways of working, fluctuated depending on individual respondents, e.g., some respondents reported an increase in stress over time, while this appeared to decrease for others.

### Dental care professionals

Seventeen dental nurses, 1 therapist, and 7 hygienists completed diary entries between July and December 2020. From these entries, 20 were considered valid (14 dental nurses, 1 therapist, 5 hygienists).

#### Career concerns

Amongst the DCP respondents, a range of concerns related to the impact of COVID-19 on their careers were raised. These included fear about their short to medium term job security and associated financial concerns. Comments were also directed towards longer term career anxiety. Two hygienists stated concerns and frustrations about the uncertainty of when they would return to work after being furloughed. For one of these respondents, these anxieties increased over time, developing into concerns about their professional future:

“*It does make me feel that there is a lack of consideration for the practice hygienists and makes me wonder what the future holds*.” (Hygienist, Week 17)

Dental nurses also raised the issue of job security although no change over time was observed within the dental nurse cohort. One dental nurse expressed concern about how COVID-19 lockdowns would impact upon the future of dentistry. Worries were also raised by two respondents (one therapist, one hygienist) about reduced income, due to fewer patients attending for treatment. For the hygienist this was a constant worry, mentioned several times throughout their diary entries: no change over time was observed.

#### Working environment

Seventeen DCP respondents (11 dental nurses, 1 therapist, 5 hygienists) raised concerns related to their working environment, in particular their workload or working pattern. Related to this theme were entries regarding adapting to new ways of working, and staffing issues. Several respondents noted that they had been furloughed, with their diary entries detailing changes to their working pattern over the course of the data collection period. Entries covered anxieties about not knowing how long furlough would last, to apprehensions about returning to work. Diary entries related to workload varied in terms of how they changed over time—this was the same across the three professional roles. Some respondents experienced very little change over time, reporting either constant busyness or continually low patient numbers. Others did experience change over time, with some experiencing fluctuating workloads throughout the data collection period, and others noting a steady increase in workload over time, alongside a rising patient backlog and extra pressures on their workload. Contributing to increases in workload were issues associated with staffing—diary entries from two respondents expressed ongoing concern regarding the shortage of staff in their practices; the situation improved towards the end of their diary entries when the furlough scheme ended. Additionally, two dental nurses made repeated comments related to having to adapt to new ways of working, e.g., scheduling appointments—these comments were consistent across their diary entries and did not change over time.

Concerns related to the effects of the working environment on morale in the practice were raised by four respondents (one hygienist, three dental nurses). In all instances, these respondents were reporting low morale in their team or practice. For two respondents this situation was a constant throughout their diary entries and attributed to changes in practice management and a “*toxic*” working environment. The hygienist's concerns increased over the course of the diary entries, with comments about an unpleasant working environment changing over time to reports of low morale:

“*Very low staff morale making for a stressful and tiring day. Everyone seems to be so stressed and unhappy that they are not working well as a team. Staff feeling under pressure and struggling to cope with workload meaning that they are unwilling or unable to help and support others.”* (Hygienist, Week 22)

One dental nurse began by saying that morale was low, but over time this moved to feelings of apprehension about raising any problems or issues with anyone, and a change in practice management. By the end of the data collection period their diary entries had changed once more, to report that the team were happier, having adapted to their “*new normality*”.

#### Health and wellbeing

Two respondents (1 hygienist, 1 dental nurse) reported physical health issues as a result of their work, with one noting they had experienced shoulder and neck pain, and the other skin issues and headaches from extensive PPE use.

Five dental nurses used their diary entries to report opportunities for respite from work. Two made repeated mentions of using their annual leave as a way of getting away from work and two respondents noted that they felt better after taking a break from work.

The negative impact of the pandemic on mental health and wellbeing was noted by several respondents. For some these were one-time reflections. However, others reported regularly on their experiences of stress and anxiety. One respondent writes several consecutive diary entries detailing constant low mood and the impact of irregular and unpredictable workload, increased use of PPE and changes to AGP delivery as all affecting the respondents' day-to-day physical and mental health:

“*By the time you leave to go home you are done, exhausted mentally, struggling to find the motivation to do anything other than go home and sleep.”* (Dental Nurse, Week 17)

Box 3 Lesley’s* Story.

Lesley is in her late 40s and working as a Hygienist in a mainly private practice. Her first diary entry is on 28th August. She is the only hygienist with the practice to have been taken off furlough and has just returned to clinical work for 2 out of her contracted 3 days; she does not know when she will be able to resume her full hours. The appointment books are full, she is working 5-hour sessions without “having a chance to even get a sip of water or a loo break” and running late due to the extra time needed to call patients in from the carpark, take their temperature, provide hand rub, etc. She is very concerned about the coming weeks, there are not enough staff to do everything (which adds to the delays) and the pace of work for her, the nurses and the reception staff is too much: “I do not feel anyone could work like this long term.”

As the weeks pass Lesley continues to struggle with her working conditions. She continues to run late, is sometimes without nurse support, all of which puts more pressure both on her and on the appointment book. The removal of blinds and the requirement to have the patio doors open “means when it is cold, we are very cold. When it is hot, we are scorched”. She eats lunch in her car to keep safe, as she feels that implementing all the distancing guidelines in the building is impossible. She suffers with pain and cramps whilst hand scaling. Patients who were cancelled March-August have not been contacted with new appointments, yet she is seeing 6/12 monthly recalls and some new patients. She feels this is unfair, but her concerns are dismissed. There are also ongoing tensions between the company, the practice management and the wider staff which adds to the overall confusion and frustration felt amongst the team: “We do not know who to listen to and what advice we should be following.” Lesley has to refuse when one of the dentists tries to book her for an AGP appointment and she struggles to explain to patients why she cannot carry out a Cavitron scale for them but a dentist can. Work is hectic and as Covid numbers rise “there is no light at the end of the tunnel which is worrying”.

23rd October and furlough ends tomorrow. Lesley has still not been informed of her position regarding her part furlough status and does not know what her income will be next week. She is concerned the company may reduce Hygienist hours: how much work will there be for her in the future? Meanwhile the practice continues to be short staffed and has been unable to attract a new dental nurse. Furlough is extended but she receives no direct communication on the matter and must assume she is still on part furlough as previously. Working flat out all day with a short 10–15 min to eat lunch only and doing unpaid overtime to catch up on notes and prepare has become the norm.

As December approaches the receipt of a (not unwelcome) Christmas bonus cannot address the underlying issues or alleviate the enormous pressures felt by Lesley and her colleagues and by mid-December staff morale is “at an all-time low” resulting in “petty arguments” and an “unpleasant atmosphere”. Team members are so overwhelmed with the workload and pressure they seem unable and unwilling to help one another. Personal time has to be used to read emails and watch webinars which provide essential information from the parent Company. In her final diary entry on 18th December Lesley reflects on the previous six months how “I certainly thought the situation would have improved by this time and the thought of more months of working like this is very daunting”.

*Lesley is a pseudonym.

This respondent experienced shame about how they are feeling:

“*It feels almost like no one else is worried, my work colleagues either seem to be coping better with it or they don't care so much about it all, which makes me feel a bit stupid, like I'm overreacting.”* (Dental Nurse, Week 18)

Their mood improves towards the end of the data collection period, attributed to an increase in their antidepressant medication.

Three respondents, all dental nurses, used their diary entries to detail their positive experiences, with all three making repeated mention of having a good week at work, with one noting they were “*maintaining ‘we can do this’”*.

Two dental nurses noted the impact of difficult personal news on their anxieties and concerns. In both instances the personal news was related to the health of a close family member. For one respondent this was two separate instances with no change over time, but for the other respondent, the stress of their family situation resulted in constant stress throughout their diary entries, at one point reporting having “*a breakdown*” on a Saturday due to ongoing stress with work and home life.

#### Support from management and colleagues

The importance of a supportive work environment emerged clearly from the data. Respondents noted how they were supporting other members of staff, support they were receiving from practice management, as well as feelings and experiences of teamwork within their practice. However, there were also diary entries that detailed disagreements and complaints about management, and tensions with senior members of staff.

Regarding offering support to colleagues, this was noted by three hygienists, where it was linked to staff morale, and presented as a consistent concern. One dental nurse discussed being supported by their team, with later diary entries describing how they returned this support to colleagues particularly in times of stress.

One hygienist's diary presents expressions of frustration with their practice management in their early entries, these changing to a more positive and optimistic outlook by the end of the data collection period when they had been given a Christmas bonus. One dental nurse described constant pressure and lack of support from practice management throughout their diary entries; they described this as a “*constant battle*”. A second dental nurse also noted ongoing disagreement with their management over a five-week period on the topic of AGP rules.

#### COVID-19

Challenges regarding COVID-19 precautions in practice were raised by nine respondents (4 hygienists, 1 therapist, 4 dental nurses). Where challenges were raised in more than one diary entry, these related to ongoing issues affecting respondents, with no change over time. Most concerns were related to PPE, but others detailed their frustration with rule changes and the need for open doors and windows for ventilation purposes:

“*I am working with open patio doors all day as my employers insist this is a requirement for ventilation despite not carrying out AGPs… We are now arriving at work in frosty conditions and having to work with the doors open with wind and rain coming in. The doors open right by the patient's head. This is not an acceptable situation.”* (Hygienist, Week 18)

Seven respondents noted in their diaries concerns related to Covid-19 more generally (one therapist, 6 dental nurses). These issues were related to rising Covid-19 levels, risk of infection, and worry about future lockdowns. For some, a fairly consistent level of stress and anxiety was expressed across their diary entries, but for others their concerns did change over time, for instance with the prospect of vaccination towards the end of data collection. For some this prompted feelings of relief, but for one person this led to an increase in anxiety, related to concerns about the safety of the vaccine.

#### Patients

Seven respondents (1 hygienist, 6 dental nurses) used their diary entries to express a variety of concerns related to dealing with patients. While for three respondents (all dental nurses) this was a one-off mention about an aggressive patient or frustration related to having to explain to patients what treatments were available, for the other four respondents dealing with patients was a constant concern expressed throughout their diary entries. Issues included: the impact of the pandemic on patients and their expectations, i.e., increased fees, waiting lists, and level of treatment, and, most crucially, the impact on patients' dental health. This patient-focused content was a constant feature over the data collection period and was associated with feelings of guilt about not being able to treat patients.

“*Phone calls are constant with patients wanting [to be] seen and we are still very limited of how many we can see and what we can do… [I] have great empathy for them and in a way [I] feel they are being badly let down.”* (Dental nurse, Week 11)

#### Change over time

Diary entries from the DCP group showed a great deal of variation between individuals. For example, with regard to low team morale within the practice, over time this remained constant for two, worsened for one, and improved for another. Similarly, concerns related to Covid-19 reduced over time for one DCP but increased for another. Concerns related to job security, workload, adapting to new ways of working, and dealing with patients remained constant throughout the data collection period, demonstrating that the impact of the pandemic resulted in anxieties and concerns which were sustained over several months.

## Discussion

The aim of this study was to explore, qualitatively, the themes emerging across and between different groups of staff and how these changed over time. There were several themes that emerged in all three professional groups: job security; the need to adapt to new ways of working or changes to the working environment, including fluctuating workloads; anxieties about COVID-19; the benefits of respite; the impact of the pandemic on practitioners' health and wellbeing; and the impact of restrictions on patients and their oral health.

While there were similarities between the themes across professional groups, the trajectories differed for some, as did the causes of, and nuances within, the themes. While all groups discussed their increasing concern about the impact of the pandemic on their career, and their job security, only dentists reported the issue of the impact on their dental practice as a business, and consideration of leaving the profession. Dentists and DCPs expressed concerns about the future of dentistry, but this concern was not apparent within the trainee group. The trainees were grappling with issues unique to their group, namely the impact of the pandemic on their training experience and sufficient opportunities to fulfil curriculum requirements. Similarly, while all groups discussed adapting to working with PPE and other COVID-19 precautions, this appeared to be an ongoing challenge that did not change over time for the DCP group, whereas concerns fluctuated over time for the trainees and dentists, with more variation found within these groups. The impact of the pandemic on respondents' wellbeing also seemed to remain constant, or increase, for all three professional groups. The experience of workload differed between the groups: some DCPs discussed being furloughed while others reported either very busy or very quiet workloads; trainees had initially low workloads, although this did increase over time; and dentists reported a constant or increasing level of busyness. While the topic of feeling supported did emerge from all three professional groups, the experiences of support differed. Trainees noted the benefits of support from their supervisors and colleagues, and this emerged repeatedly in their diary entries, whereas the only mention of support from dentists was in relation to the perceived lack of support from health boards or the GDC. Only the DCP group stressed the importance of being supported and supporting others.

The impact of restrictions on relationships with patients and their oral health was an important theme across all groups, and a topic that also emerged from data collected from focus groups (18) and the survey (5) conducted as part of the wider CAREER programme of research, illustrating that the impact on patient care during the pandemic was a significant concern for all dental professionals. For some respondents, this elicited feelings of guilt about not being able to provide the level of care that they felt patients should receive; indeed, one respondent referred to this as a moral dilemma. In a scoping review, Čartolovni and colleagues noted that the COVID-19 pandemic added to the existing burden of healthcare professionals by inducing a sense of “dissatisfaction with the care provided to their patients”, which they believed could lead to feelings of guilt or moral injury (19). Moral injury has been described as an emotional wound caused by challenges to an individual's moral or ethical code; it is also considered to be one of biggest potential long-term impacts of the pandemic on healthcare professionals (19, 20). Čartolovni and colleagues posit that if a healthcare professional experiences such challenges over a period of time—as the CAREER diaries demonstrate—this could begin to impact upon their daily practice, which in turn could affect the level of care they are able to provide to their patients. These authors also identify that moral injury, and the related construct of moral distress, can result in reduced job satisfaction, considerations about leaving the profession, and burnout; they suggest that healthcare professionals could develop burnout if the impact of moral injury is not addressed “in a timely manner” (19).

Burnout has been found to contribute to career disengagement. Indeed, the issue of disengagement was present in the diary study, with some respondents revealing their thoughts about changing roles, retiring early or leaving the profession altogether. A recent study found that physicians with burnout were three times more likely to have thoughts about quitting their jobs, with emotional exhaustion a significant contributor (21). Physician burnout caused by high workload, staffing issues, a lack of support, leadership issues, and moral injury, has also been described as a risk to patient safety (22). All these factors have been evidenced in the diary study, as existing and co-existing over time, suggesting that respondents experiencing repeated ethical or moral dilemmas, particularly related to the care they are able to provide to their patients, are susceptible to developing burnout. This argument supports the view that those in leadership and management positions within dentistry need to consider not just the short to medium term, but also the longer-term impacts of the pandemic on their staff (20).

We believe that this is the first instance of a longitudinal diary study of dental trainees and primary dental care staff in Scotland. This qualitative study has allowed for a deeper exploration of the factors influencing dental practitioners' anxieties and concerns during the COVID-19 pandemic, by using respondents' diary entries to highlight issues as they occurred in real time, and not retrospectively as with focus groups and interviews. Using a trajectory analysis provided a unique opportunity to explore how experiences and responses changed over time, complementing the earlier work of Freeman et al. (6). This study design of offering frequent open-ended opportunities to share some important inner focused beliefs and feelings was revelatory as the method enabled the possibility to detect changes that would not have been obvious if using a repeated cross-sectional analysis (23). An important example to illustrate this was the respondent who was able to outline the deterioration in their mental health status and eventual “breakdown”, as they described it. It is important to highlight that both the qualitative (reported here) and the quantitative (6) trajectory analyses confirm a large variation of reactions in response to the pandemic over the six-month period. It is notable that stable high levels of pressure were being reported as well as increasing and decreasing reactions to events and circumstances during working practice and conditions experienced by respondents. Of particular interest is the support of the face validity of the qualitative methodology employed as indicated by the sensitivity and high level of disclosure of changes to emotional life within the work setting.

The diary methodology enabled not only the example of distressing unravelling of mental health, but also examples of staff adapting to the pressures posed by the pandemic, either through direct experience gained, as reported by some trainees, or through a proportion of qualified staff simply learning to accept change, almost as a natural process. Analysis of individual and group trajectories revealed the immediate and longer-term factors affecting respondents, highlighting issues that remained ongoing, as well as those whose significance fluctuated or developed over time. This suggests that any future interventions should consider the short- and longer-term needs of practitioners when developing “suitable and ongoing ways” to improve the mental health and wellbeing of the dental profession (24). Knights and Humphris have made several suggestions regarding what future interventions could look like, including a need for the development of a suitable long-term post-COVID-19 NHS funding model for UK dentistry. They also emphasise the importance of further developing the evidence base needed for effective intervention and propose a proactive approach to the ongoing assessment of the mental health and wellbeing of the dental profession (24).

There are several inevitable limitations to this study. The diaries were self-reported and completed by practitioners who volunteered to participate, having already taken part in the initial baseline CAREER survey (5). As such, the data may be affected by selection bias and although the findings could be applicable to other contexts, situations, and populations, they should not be considered as directly generalizable to the wider dental profession. While diary respondents were given the prompt to use the free text box if they had any significant issues or concerns because of the impact of Covid-19 on their work, it is possible that issues raised in the diary entries were not directly associated with the pandemic. In addition, the completion of diary entries by participants was often inconsistent, with some completing every entry over the data collection period and others having multiple gaps, sometimes of several weeks.

In summary, the honesty and candor with which respondents completed their diary entries revealed a great deal of uncertainty and anxiety related to their day-to-day working lives, their training, and their professional futures. The use of longitudinal diaries served to highlight the significant emotional toll that the pandemic has had on the dental profession. This study, alongside the findings from previous CAREER publications, illustrates the acute need not only to ensure individual practitioners are able to access timely and effective mental health support, but also for appreciable changes to be made at departmental (team) and organisation levels to support the mental health and wellbeing of dentists, along with policy and system-wide interventions (24). As demonstrated by the findings of this study, support needs to be tailored and flexible to the short- and longer-term needs of practitioners, recognising that anxieties and concerns can fluctuate over time.

## Conclusion

These findings have demonstrated the need for increased provision of mental health and wellbeing support services for dental staff. Future interventions should take into consideration the fluctuating nature of dental professionals' concerns and anxieties over time, to provide support that can address both immediate and ongoing issues.

## Data Availability

The datasets presented in this article are not readily available because the ethical review board instructed that the data collected remain within the NHS Education for Scotland repository and jurisdiction. Requests to access the datasets should be directed to sdpbrn@nes.scot.nhs.uk.

## Footnotes

^1^PCT diaries were open from 4th September to 18th December 2020. Trainee diaries were open from 10th July to 18th December 2020. Trainee dental nurses and TOTs diaries were open from 18th July to 18th December 2020.
